# Current Trends and Outcomes for Open vs. Arthroscopic Latarjet

**DOI:** 10.1007/s12178-024-09889-9

**Published:** 2024-03-11

**Authors:** Filip Vuletić, Berte Bøe

**Affiliations:** 1 Department for Orthopaedic and Trauma Surgery, University Hospital “Sveti Duh”, Sveti Duh 64, 10000 Zagreb, Croatia; 2 Faculty of Kinesiology, University of Zagreb, Horvaćanski zavoj 15, 10000 Zagreb, Croatia; 3 Division of Orthopaedic Surgery, Oslo University Hospital, Trondheimsveien 235, 0586 Aker, Oslo Norway

**Keywords:** Shoulder instability, Latarjet procedure, Arthroscopic Latarjet, Clinical outcomes, Complications, Return to play

## Abstract

**Purpose of Review:**

This paper aims to analyze and compare the existing research on open and arthroscopic Latarjet procedures for treating anterior shoulder instability. The review will assess different factors such as graft positioning, functional outcomes, complications, and return-to-play rates for both approaches. The study’s primary goal is to establish which technique yields superior outcomes.

**Recent Findings:**

Recent studies have suggested that arthroscopic Latarjet surgery can produce outcomes similar to open surgery regarding functional scores and patient satisfaction. Some research indicates that arthroscopy may even provide slightly better results. Both techniques have similar complication rates, but arthroscopy requires a longer learning curve and operating time. It is crucial to ensure the proper placement of the graft, and some studies suggest that arthroscopy may be better at achieving accurate positioning.

**Summary:**

Both open and arthroscopic Latarjet procedures are equally effective in treating shoulder instability. While arthroscopy offers a faster recovery time and causes less soft tissue damage, it requires surgeons to undergo a steeper learning curve. The optimal graft position for both techniques is still debated. More long-term data is needed to establish superiority. Future research should compare approaches in larger cohorts and identify outcome-affecting factors to improve the treatment of shoulder instability. Both techniques are promising, but arthroscopy may be a better option as the procedure evolves into a less invasive reconstruction.

## Introduction

Over the years, various surgical techniques have been developed for treating anterior shoulder instability. The Latarjet procedure stands out as a highly effective solution typically recommended for patients at a higher risk of postoperative recurrences ^1^. Such patients are those experiencing their first dislocation at a young age, engaging in high-risk sports, exhibiting prolonged instability, enduring severe glenoid bone loss and hyperlaxity, or who have previously undergone an unsuccessful soft-tissue Bankart repair [1]. The Latarjet procedure as a primary procedure has significantly better outcomes for postoperative instability than an arthroscopic Bankart repair, with rates of 3% and 28.4%, respectively [2, 3]. Although the Latarjet procedure can restore stability, some surgeons prefer to use it as a secondary option after failed arthroscopic soft tissue stabilization. Recent studies have presented conflicting findings regarding the use of the Latarjet procedure as a revision surgery compared to its use as a primary treatment for anterior shoulder instability [4]. While some publications suggest that a revision Latarjet surgery after an unsuccessful labral repair may result in better outcomes and, therefore, could be a more appropriate primary surgical choice, other studies have found no significant difference between primary and revision Latarjet repair outcomes [5, 6]. Further research is necessary to determine the optimal use of the open Latarjet procedure in different clinical scenarios and compare the outcomes to advancements in arthroscopic technique.

The Latarjet procedure was first described in 1954 as a surgical solution for treating shoulder instability [7]. It stabilizes the shoulder through the transferred coracoid process’ static effect and the conjoint tendon’s dynamic sling effect. Correct coracoid process placement and fusion are crucial for successful surgery outcomes [9, 10]. Several biomechanical and clinical studies have shown that precise graft positioning can significantly affect the procedure’s outcome [9, 10]. There are some concerns regarding the optimal placement of the bone block, which can result in clinical complications. Complications of the Latarjet procedure include recurrence of instability, loss of external rotation (which can make it difficult to return to competitive sports), failure of bone union, infection, nerve injury, hardware removal, and the development of arthritis in the long term [10]. Studies show that the open Latarjet procedure gives excellent long-term outcomes in treating anterior glenohumeral instability, with a postoperative recurrence rate of only 5.9% in a 20-year follow-up [5]. The arthroscopic Latarjet, a newer, minimally invasive technique, has gained popularity among physicians [11]. It offers advantages, such as decreased stiffness and quicker rehabilitation [11, 12]. Some studies have shown that the arthroscopic Latarjet procedure leads to similar clinical results compared to the open surgery after short- and mid-term follow-ups [13–15]. However, the long-term outcomes of the arthroscopic Latarjet procedure have yet to be thoroughly studied [16•]. While the open Latarjet technique is more invasive and may entail prolonged recovery times and increased pain [17], the arthroscopic approach is less invasive and may result in shorter recovery times and less pain [12]. Nonetheless, it presents technical challenges and may be applicable only for selected cases of shoulder instability [18]. As expertise grows and guided systems emerge to enhance reproducibility and minimize complications, the arthroscopic technique continues to refine. The evidence supporting the effectiveness of arthroscopic performance is growing, and it is now generally accepted that both techniques can effectively treat anterior shoulder instability.

## History and Evolution of Open and Arthroscopic Latarjet

**Fig. 1:**
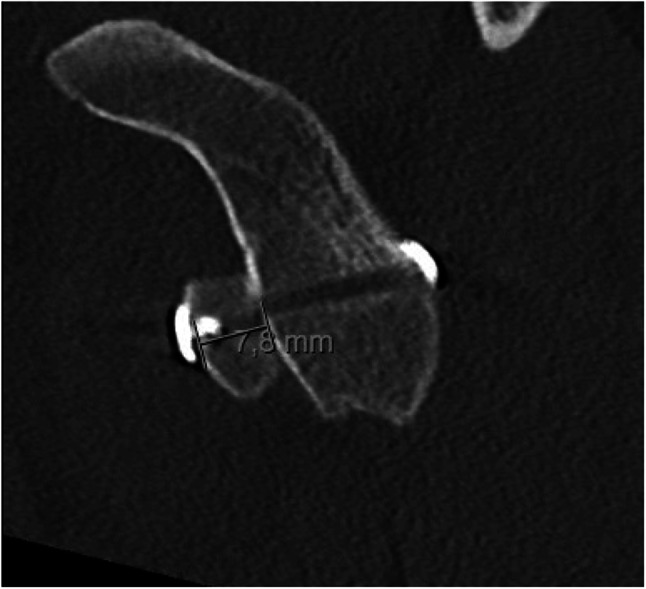
Fixation of the coracoid graft with double-button fixation in arthroscopic Latarjet procedure

Michel Latarjet introduced an open technique for treating anterior shoulder instability in his 1954 publication “Treatment of Recurrent Dislocation of the Shoulder” [7]. The procedure garnered significant interest due to its promising outcomes. Over time, the open Latarjet procedure has undergone significant evolution. These changes include refining the surgical approach, optimizing graft placement and fixation, and enhancing stability. From the original extensive deltopectoral approach, surgeons have adopted smaller incisions and limited dissection, reducing pain and faster recovery [19]. The management of the subscapularis tendon during the open Latarjet procedure has evolved. Initially, a complete subscapularis tenotomy was performed to gain better access to the glenoid [7]. However, this approach resulted in suboptimal functional outcomes and increased the risk of postoperative shoulder stiffness [20]. Subsequently, procedures were modified to preserve the subscapularis tendon, such as limited (split) tenotomy, to maintain tendon integrity and improve postoperative shoulder function [20]. Central to procedural success is the precise positioning of the coracoid graft, which is essential for restoring glenoid anatomy and mitigating recurrence risks [9, 10]. If the bone block is placed too medially, it could result in a higher recurrence rate [21]. Conversely, setting the graft too laterally is associated with a greater incidence of degenerative changes [22•]. Even so, in a radiographic evaluation study by Hovelius et al., incorrect graft positioning occurred at a rate as high as 67% in the open technique [23]. Similarly, Walch et al. found that a significant number of coracoid bone blocks were improperly transferred during the procedure [24]. Specifically, 27% were moved too far laterally, while 12% were transferred too medially [24]. The proper screw placement is also proven to be critical [25]. Lädermann suggested that it is best to place screws within 10° of the glenoid articular surface to avoid intruding on the humeral head and injury to the suprascapular nerve [26]. This can be achieved by keeping the screws parallel to the glenoid surface. However, concerns regarding screw loosening, graft migration, and postoperative osteolysis have led to the exploration of alternative fixation methods. These modifications include suture anchors and double-button fixation systems (Fig. 1), which aim to improve coracoid graft stability and minimize complications [27].

In 2007, Lafosse et al. introduced the first arthroscopic Latarjet technique to reduce the open approach’s invasiveness and complications [28]. This technique involves coracoid transfer using two screws for fixation and provides a less invasive approach with potentially improved visualization compared to the open procedure [28]. Subsequent studies have focused on refining the arthroscopic Latarjet procedure. In 2010, Lafosse et al. published results collected from the first 100 cases demonstrating the feasibility and safety of the arthroscopic technique [12]. They emphasized the importance of proper patient positioning, portal placement, and graft fixation techniques for successful outcomes [12]. Several modifications have been proposed to enhance the reproducibility and reliability of the arthroscopic Latarjet procedure. One notable technique pioneered by Pascal Boileau involves coracoid fixation utilizing endobuttons. This method incorporates tension devices, specialized guides for posterior tunnel drilling, double-loaded suture anchors, double-row fixation, and supplementary stabilization techniques like capsular plication or remplissage [27, 29•].

Advancements in arthroscopic technology and surgical techniques have made it possible to use the arthroscopic approach with positive clinical outcomes, which will be described further in the text. However, additional research is required to establish its long-term effectiveness.

## Outcomes of Open and Arthroscopic Latarjet

**Fig. 2:**
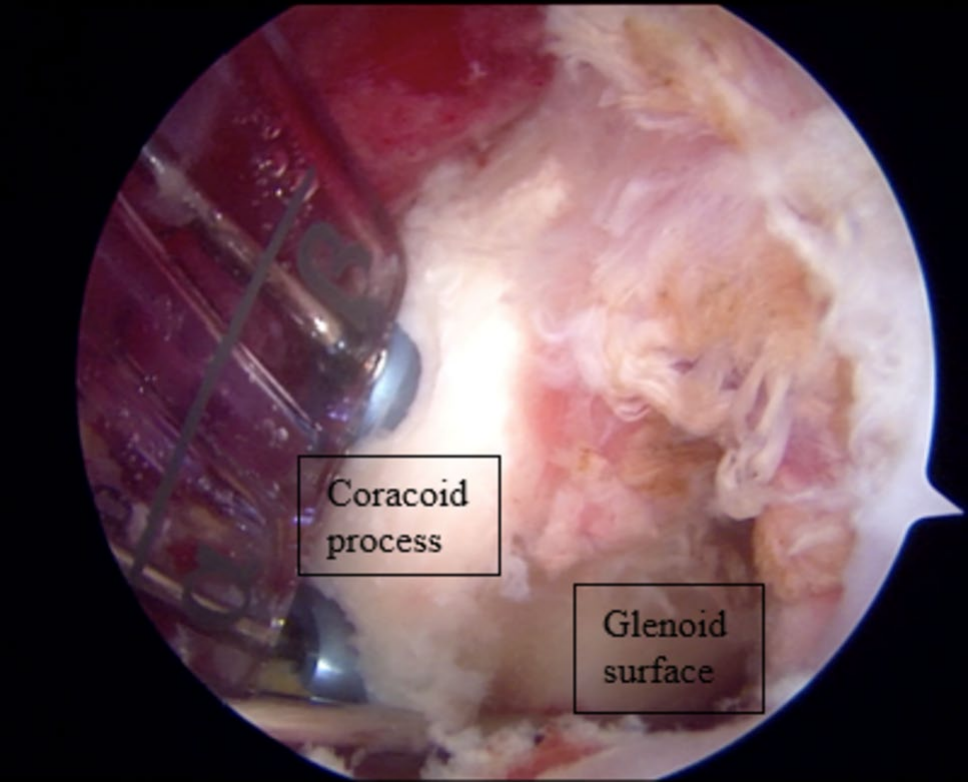
The fixation of the coracoid graft with two screws onto the glenoid during the arthroscopic Latarjet procedure

Accurately placing and securing the coracoid bone block is crucial for the success of both open and arthroscopic Latarjet procedures [9, 10]. The primary goal is to reconstruct the anteroinferior glenoid, which is susceptible to erosion or fracture, in patients with recurrent instability [30]. Open surgery may make visualizing the glenoid more difficult, but it simplifies some technical steps [12]. On the other hand, the arthroscopic technique enables visualization of the complete glenoid articular surface, potentially reducing the risk of incorrectly positioning the graft (Fig. 2) [12, 28]. Controversy exists in the literature regarding whether the open or arthroscopic procedure is superior for graft positioning. According to research conducted in 2017, patients who underwent the open Latarjet procedure showed better results in positioning the graft on the coronal plane than those who underwent the arthroscopic procedure [31]. While Kordasiewicz et al. found no significant difference in the appropriate bone block position between the two groups, Ali et al. observed a higher incidence of lateral graft positioning in open surgery [25, 32]. While the proper mediolateral positioning of the graft has clear guidelines in the literature, the ideal superior-inferior placement of the coracoid graft is still a matter of debate among experts [33]. Different authors suggest various positions, some recommending placement below the equator or below 3 o’clock, while others propose slightly different positions [33]. Casabianca et al. suggest the perfect position is between 2:30 and 4:20, while Lafosse and Boyle recommend a vertical graft position between 3 and 5 o’clock [12, 34]. Recent studies suggest that open and arthroscopic Latarjet procedures yield comparable results, but there is a significant difference in the vertical positioning of the graft [11, 25]. According to one study, the arthroscopic procedure resulted in a significantly lower equatorial position compared to the open procedure [13].

When fixing a graft, it is important to correctly position the screws to avoid complications. A study was conducted to investigate the distance between the exit point of screws used to secure the coracoid graft and the suprascapular nerve during the Latarjet procedure. The study found that the average distance between the posterior exit point of the superior screw and the suprascapular nerve at the base of the scapular spine was only 4 mm [26]. As mentioned earlier, the screw should be angled no more than 10° from the glenoid surface (known as the alpha angle) to ensure proper and safe placement [26]. Although studies have found that the *α* angle for both the arthroscopic and open Latarjet techniques falls within the currently accepted limits, suggesting that the difference may not be clinically significant, a slight improvement in screw positioning has been reported in the arthroscopic approach [25].

### Radiological Findings

In their study, Giacomo and colleagues discovered that graft osteolysis can occur up to 63.9% of the time, making it one of the most frequent complications of the procedure [35]. Ali et al. found that resorption was more common after open surgery than arthroscopic surgery [32]. They discovered a link between graft resorption, monitored through postoperative computed tomography (CT), and a positive apprehension test [32]. However, the correlation between graft resorption and other functional tests was not observed [32]. According to the literature, radiological findings that suggest graft resorption may have little clinical significance [10, 35]. Conversely, recent studies have indicated a significant correlation between fractures in the coracoid graft and clinical outcomes [36]. Kordasiewicz et al. discovered that open surgery leads to a greater chance of postoperative graft rupture and bone union issues when compared to arthroscopic techniques [25]. A recent study discovered that intraoperative graft fracture was similar in open and arthroscopic Latarjet procedures [37••]. However, a comprehensive review found that there were more cases of intra- and postoperative graft fractures in patients who underwent arthroscopic treatment as compared to those treated with the open Latarjet procedure [11]. Hence, there is room for an arthroscopic technique to improve crucial steps in preventing graft fractures.

Since its development, numerous studies have demonstrated the safety and reproducibility of arthroscopic Latarjet despite being described in the literature as complex with a longer learning curve [12, 38].

### Functional Outcomes

Minimally invasive procedures like arthroscopic Latarjet surgery are less painful and have better functional outcomes in the early postoperative period compared to open surgery [39]. However, both techniques have been found to yield similar clinical outcome scores in the longer-term follow-up [13, 40]. One study assessed the functional outcomes of arthroscopic and open Latarjet procedures using scoring systems, such as the Constant-Murley score and American Shoulder and Elbow Surgeons (ASES) score [41]. The results have been generally favorable for both techniques [41]. In 2021, a group of researchers led by Hurley conducted a retrospective analysis of patients undergoing either arthroscopic or open Latarjet procedures [42••]. The researchers measured various factors, including visual analog scale (VAS) score, Shoulder Instability-Return to Sport after Injury (SIRSI), Subjective Shoulder Value (SSV), Western Ontario Shoulder Instability (WOSI) score, patient satisfaction, and willingness to undergo surgery again [42••]. Their results showed no difference in functional outcome scores or recurrence rates between the two approaches [42••]. Ali et al. discovered a significant difference in the WOSI score between the two techniques, which met the previously published minimal clinically important difference (MCID) and favored the arthroscopic technique [32]. In a prospective clinical study, Nourissat et al. reported slightly better functional outcomes in the arthroscopic Latarjet group [40]. Several studies have compared the range of motion outcomes between arthroscopic and open Latarjet procedures. Zhu et al. reported similar external rotation and forward flexion between the two techniques [41]. Ali et al. noted a difference in internal rotation loss that favored the open Latarjet [32]. However, it is essential to note that the differences in the range of motion were not clinically relevant. In the literature review conducted by Hurley et al., both approaches yielded satisfactory results and similar ranges of motion after surgery [44].

This suggests that both techniques have favorable functional outcomes, with some studies suggesting slightly better outcomes with the arthroscopic approach.

### Complications

Studies have shown that the rate of complications is similar for arthroscopic and open Latarjet procedures. Although there were concerns that the arthroscopic approach could result in higher complication rates and ultimately lead to higher revision rates, Hurley et al. found no significant difference between the open and arthroscopic approaches [42••]. A multicenter analysis reported a less than 10% complication rate for arthroscopic Latarjet cases and 3.8% of patients needing additional surgery [43]. Similarly, a study by Hurley et al. reported a 6–7% complication rate for open Latarjet cases, with the most complications being graft-related [44].

It was found that the Latarjet procedure had a meager recurrence rate overall, with only 2.2% of patients experiencing postoperative dislocation. Studies have shown that there were no significant differences in the postoperative recurrence rate between the open and arthroscopic Latarjet procedures [13, 42••].

**Fig. 3:**
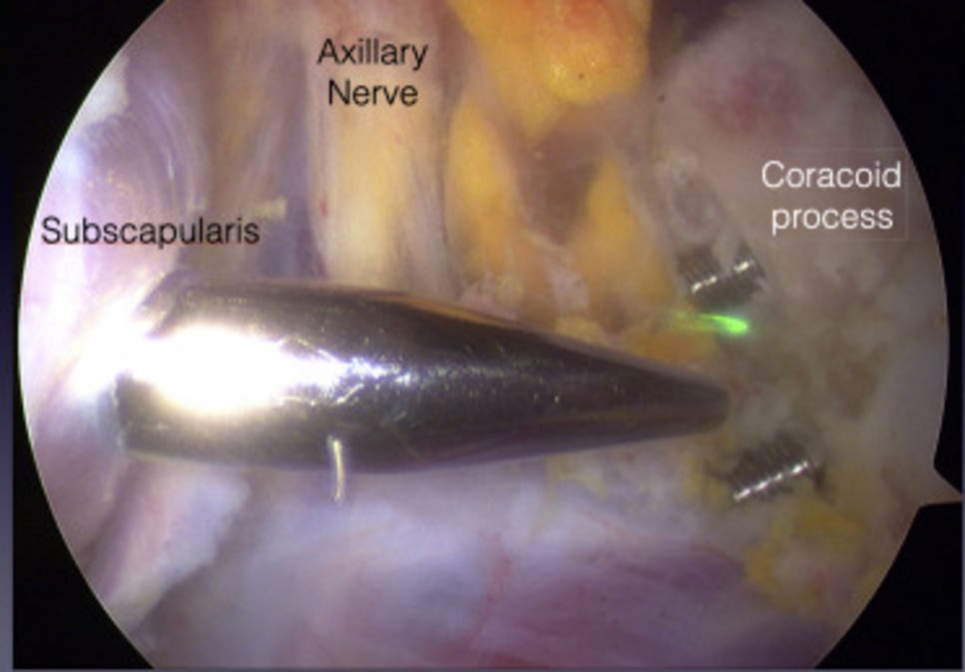
Arthroscopic visualization of nerve structures can minimize potential iatrogenic injury (courtesy of Bøe et al. 2022)

Nerve injuries are rare in both arthroscopic and open Latarjet procedures. In a prospective study by Zhu et al., they observed no instances of nerve injury in either open or arthroscopic Latarjet procedures [41]. Two studies led by Boileau have shown that no nerve damage was identified after the arthroscopic surgery [45, 46]. Lafosse’s group conducted a 5-year follow-up study that revealed only one patient out of 64 (1.6%) experienced muscle wasting of the anterior deltoid [47]. Some may argue that the low rate of complications is due to highly experienced surgeons performing the arthroscopic Latarjet procedure. However, studies assessing the learning curve’s impact on complications have also found limited rates of nerve injuries [30, 48••]. Before performing the subscapularis split in an arthroscopic procedure, examining the axillary nerve is crucial, and the musculocutaneous nerve is recommended (Fig. 3). This is not a typical step in an open Latarjet procedure, which does take additional time but enhances safety [48••].

Both arthroscopic and open Latarjet procedures have a low incidence of infection. Metaise et al. reported two infections in the open group and four in the arthroscopic group, whereas Hurley et al. found no significant difference in infection rates between the two techniques [42••, 49].

### Operative Time

The studies consistently show that the more experience surgeons have with arthroscopic Latarjet procedures, the less time they need to complete the operation. Kany et al. prospectively evaluated 104 patients and compared surgical duration between their first and last 30 patients, which decreased from 103 to 76 min [50]. Cunningham et al. demonstrated a reduction in operative time for arthroscopic Latarjet from 183 to 150 min after ten cases, then to 95 min after another ten cases, comparable to their open Latarjet duration [39]. However, Zhu et al. noted that even with experience, the arthroscopic Latarjet procedure still took around 30 to 40 min longer on average compared to the open procedure [41].

In 2022, Bøe et al. conducted a thorough prospective study to evaluate the learning of the arthroscopic Latarjet procedure [48••]. The study aimed to identify differences in early clinical outcomes, surgery time, and complications as surgeons gained experience. The researchers concluded that the procedure has a learning curve, with the initial longer operating times and higher rates of complications [48••].

### Return to Play

The ability of athletes to return to play postoperatively (RTP) following shoulder instability surgery is crucial. Arthroscopic and open Latarjet procedures have similar RTP rates. A systematic review by Abdul-Raddoul et al. found that the arthroscopic Latarjet procedure takes longer to return to sports, with a mean duration of 5.9 months, compared to open Latarjet, which takes 5.07 months [51]. However, the arthroscopic Latarjet procedure has a higher rate of returning to sports at 94%, compared to the open Latarjet procedure with a lower rate of 83.6% [51]. Meanwhile, Hurley et al. observed no significant difference in the overall rate of return to play between the two groups [42••]. However, there was a slight difference in the timing of RTP by 1 month, which favored the arthroscopic technique [42••]. According to Brzoska’s research, the arthroscopic Latarjet procedure was equally effective for all types of sports [43]. This was not true for other procedures in previous studies where overhead athletes had an RTS incidence of 76.8%, while collision athletes had an RTS in 90% of cases [43, 44].

### Cost Analysis

In 2016, Randelli et al. compared the costs of open and arthroscopic Latarjet procedures. They found that the arthroscopic technique had higher direct costs [14]. However, arthroscopic Latarjet could potentially offer reduced postoperative pain, decreased analgesic requirements, and cost savings [30]. However, no study has yet combined and evaluated both techniques’ direct and indirect costs. Therefore, further research must provide more comprehensive and reliable cost-effectiveness data.

## Conclusion

The Latarjet procedure is an effective treatment for anterior shoulder instability. The open approach has been used for more than 60 years and has undergone substantial advancement, resulting in better outcomes and fewer complications. The arthroscopic approach is a newer and less invasive technique that has recently gained popularity. Both techniques have generally good functional outcomes, with some studies indicating marginally better results with the arthroscopic approach, as shown in Tables 1 and 2. Both techniques have relatively low complication rates, including recurrence, nerve injuries, and infection. Surgeon experience plays a significant role in the outcomes of arthroscopic Latarjet procedures, decreasing operation time and complication rates as surgeons gain more experience. The range of motion outcomes between arthroscopic and open Latarjet procedures is generally comparable, albeit with subtle variations reported in select studies. Patient satisfaction rates are consistently high for both techniques. Return-to-play rates are similar between arthroscopic and open Latarjet procedures, with slightly shorter recovery periods for the arthroscopic group. The cost-effectiveness of these interventions may exhibit variability contingent upon diverse factors, including healthcare infrastructure and resource allocation.

**Table 1:** Potential advantages and disadvantages of arthroscopic Latarjet procedure

Advantages of arthroscopic Latarjet procedure	Disadvantages of arthroscopic Latarjet procedure
Smaller incisions and less soft tissue damage	Higher incidence of graft fractures
Potentially faster recovery and shorter hospital stays	Learning curve for surgeons, longer operating times during the initial phase
Comparable functional outcomes to open procedure	Higher direct costs
Potential for better visualization and graft placement	
Potential for improved cosmetic outcomes	
Potential for a better bony union and less fibrosis?	
Potential for reduced postoperative pain and analgesic requirements

**Table 2:** Potential advantages and disadvantages of open Latarjet procedure

Advantages of open Latarjet procedure	Disadvantages of open Latarjet procedure
Lower incidence of graft fractures	Larger incisions and more soft tissue damage
Comparable functional outcomes to arthroscopic procedure	Longer recovery and hospital stay
Lower direct costs	Potentially longer RTP
Potential for shorter operating times	Slightly higher risk of nerve injuries
	Potential for more noticeable scarring
